# Application of Graphene-Related Materials in Organic Solar Cells

**DOI:** 10.3390/ma15031171

**Published:** 2022-02-03

**Authors:** Lara Velasco Davoise, Ana M. Díez-Pascual, Rafael Peña Capilla

**Affiliations:** 1 Universidad de Alcalá, Facultad de Ciencias, Departamento de Química Analítica, Química Física e Ingeniería Química, Ctra. Madrid-Barcelona Km. 33.6, 28805 Alcalá de Henares, Madrid, Spain; lara.velasco@uah.es; 2 Universidad de Alcalá, Departamento de Teoría de la Señal y Comunicaciones, Ctra. Madrid-Barcelona Km. 33.6, 28805 Alcalá de Henares, Madrid, Spain; rafael.pena@uah.es

**Keywords:** graphene-related materials, organic solar cells, power conversion efficiency, transparent electrodes, active layer, hole transport layer, electron transport layer

## Abstract

Graphene-related materials (GRMs) such as graphene quantum dots (GQDs), graphene oxide (GO), reduced graphene oxide (rGO), graphene nanoribbons (GNRs), and so forth have recently emerged as photovoltaic (PV) materials due to their nanodimensional structure and outstanding properties such as high electrical and thermal conductivity, large specific surface, and unique combination of mechanical strength and flexibility. They can be a crucial part of transparent electrodes, hole/electron transport materials, and active layers in organic solar cells (OSCs). Besides their role in charge extraction and transport, GRMs act as device protectors against environmental degradation through their compact bidimensional structure and offer good durability. This review briefly presents the synthesis methods of GRMs and describes the current progress in GRM-based OSCs. PV parameters (short circuit current, open circuit voltage, power conversion efficiency, and fill factor) are summarized and comparatively discussed for the different structures. The efficiency recently surpassed 15% for an OSC incorporating polymer-modified graphene as a transparent electrode. The long-term stability of OSCs incorporating GRMs is also discussed. Finally, conclusions and the outlook for future investigation into GRM-based devices for PVs are presented.

## 1. Introduction

The conversion of solar power into electrical energy is a clean, scalable, and environmentally friendly means of energy production. Organic solar cells (OSCs) are photovoltaic devices that use organic molecules or conducting polymers to generate electricity via absorption of sunlight through the photovoltaic effect [1]. They have attracted plenty of interest over the last two decades given that they show key benefits compared to conventional devices comprising Si including their low density, elasticity, reduced production expenses, and sustainability [2]. The molecules incorporated in OSCs can be processed in solution and subsequently deposited via printing or spin-coating, allowing the production of a large volume at low temperatures and are therefore cost-effective. They also have the possibility to be transparent, enabling applications in flexible electronics, walls, windows, etc. The main drawbacks associated with OSCs are low efficiency, low stability (can experience photochemical degradation), and low mechanical resistance compared to Si-based devices [3]. In 2018, the highest efficiency for OSCs (17.3%) was attained by designing a tandem architecture [4]. 

OSCs comprise electron donor and electron acceptor materials. These are semiconductors and have a band gap that can be overcome by the electrons if they are provided with enough energy. When an electron of the donor material leaves the valence band, an unoccupied state (called a “hole”) is generated, which acts as a positively charged particle. The electron and the hole are bound together, forming a state called an “exciton”, which is explained by the Coulomb interactions between both particles. This exciton must be dissociated in order to collect the charge carries and produce an electrical current. However, this dissociation requires a specific amount of energy, which depends on the dielectric constant (ε_r_) of the material. The value of ε_r_ is a measure of the exciton binding energy, which also depends on temperature. 

**Figure 1:**
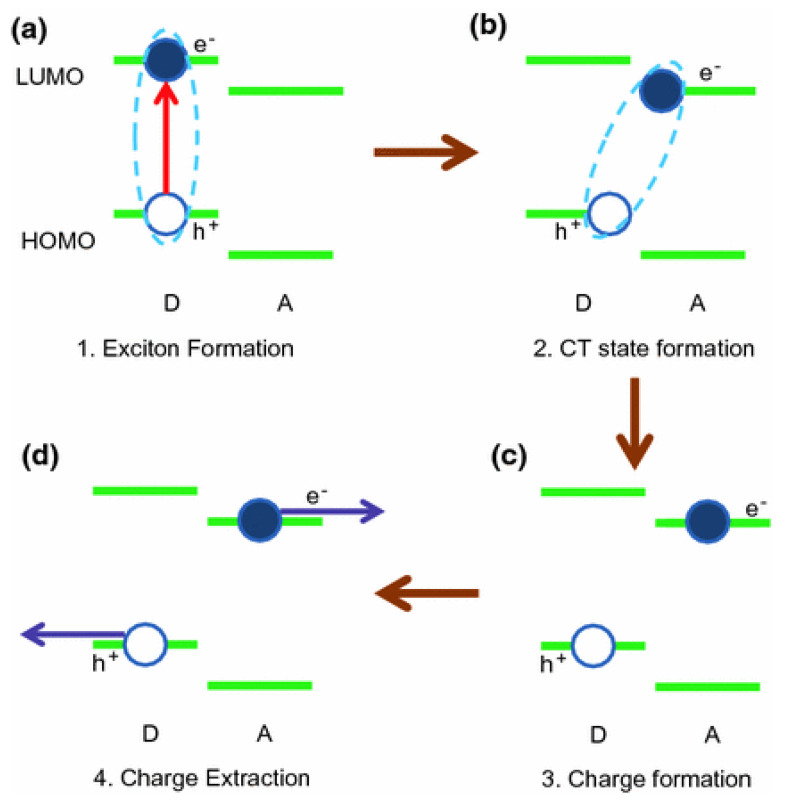
Graphic depiction of (**a**) the exciton formation, (**b**) charge transfer (CT) state formation, (**c**) charge formation and charge extraction (**d**). See explanation in the text. Reprinted from ref. [6], with permission from Springer Nature Switzerland AG.

Traditional organic photovoltaic materials exhibit low ε_r_ values, in the range of 3 to 4 [5]. The excitons are bound strongly; hence, to dissociate them, the most common approach consists of the use of materials with different band gaps. The gap depends on the difference between the lowermost empty molecular orbital (LUMO) and the uppermost occupied molecular orbital (HOMO) of each material. The junction of two materials with different LUMO and HOMO levels yields an interface called the “donor–acceptor (D–A)”: the donor material is the one with higher LUMO and HOMO values, while the acceptor is the one with the lowest values. The absorption of a photon leads to the formation of an excited state of organic material with the creation of a paired electron-hole under strong interaction forces (exciton formation, Figure 1a). The electron is transferred from the donor to the acceptor material (charge transfer (CT) state formation, Figure 1b), and the hole follows the opposite direction (charge formation, Figure 1c). After the exciton dissociation, each free charge diffuses to its respective electrode (cathode—negative charges; anode—positive charges, Figure 1d) [6]. 

**Figure 2:**
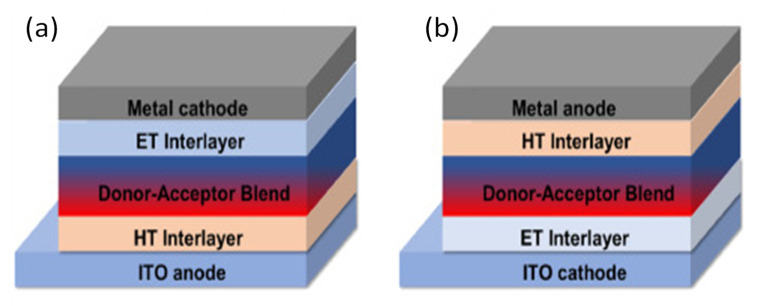
Scheme showing the structure of a typical (**a**) and inverted (**b**) OSC.

Bulk-heterojunction (BHJ) photoactive layers comprise a blend of at least two semiconductors creating a bicontinuous network of a donor (hole-transporting) and an acceptor (electron-transporting). This architecture allows a close mixing of both phases that favors exciton dissociation. The most common OSC device architectures are the “conventional” and “inverted” ones (Figure 2). In a typical OSC, the active layer is inserted between a cathode (metallic negative electrode) and a transparent anode (positive electrode), typically made of indium tin oxide (ITO). To enhance hole collection at the anode and electron collection at the cathode, a hole transport layer (HLT, i.e., PEDOT:PSS) and an electron transport layer (ETL, i.e., LiF) are used. In inverted OSCs, the cathode is usually a metal such as Ag or Au, with a high work function, while the anode is frequently ITO. 

In initial BHJ-OSCs, a conjugated polymer with a low band gap and a soluble molecule were used as the donor and acceptor, respectively, the most common being poly[2-methoxy-5-(2j-ethylhexyloxy)-p-phenylene vinylene] (MEH-PPV) polymer and fullerene derivatives such as [6,6]-phenyl-C61-butyric acid methyl ester (PC61BM). Nonetheless, the fullerene derivatives were good electron transporting materials but absorbed only a small fraction of the radiation, and the efficiency was fairly small (~7.7%) [7]. Over the last decade, high/mid-gap polymer donors such as polythieno[3,4-b]thiophene-co-benzodithiophene] (PTB7) and poly[(9,9-bis(3j-(N,N-dimethylamino)propyl)-2,7-fluorene)-alt-2,7-(9,9-dioctylfluorene)] (PFN), with some F instead of H atoms, have been synthesized. Besides, potent absorbers and low-band–gap non-fullerene acceptors (NFAs) such as rylene diimides have significantly raised the efficiencies, with values currently surpassing 13% [8]. 

**Figure 3:**
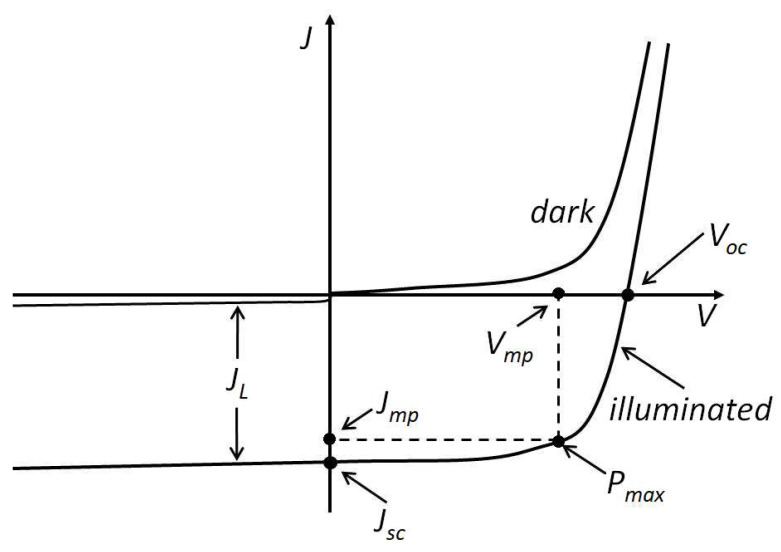
Typical current–voltage plot for an OSC. Adapted from ref. [9], with permission form IntechOpen.

The current–voltage characteristic of OSCs relates the current intensity (*J*) and voltage (*V*) in the device. By measuring these two variables, a curve such as the one depicted in Figure 3 can be obtained. The most important parameters are [9]:

- Short-circuit current (*J_sc_*): current when the voltage in the cell terminals is equal to 0 (*V* = 0). This parameter depends on the absorption of light, charge generation, transport, and extraction. These processes are temperature-dependent. *J_sc_* increases with the illumination intensity as exciton generation from absorbed phonons increase.
- Open-circuit voltage (*V_oc_*): voltage between the terminals when no current flows across the cell (J = 0). This parameter is influenced by the recombination rate of charge carriers, the work functions of the electrodes, and the energy levels of the photoactive materials employed.
- Current at maximum power value (*J_mp_*).
- Voltage at maximum power value (*V_mp_*).
- Maximum power (*P_max_*).

Two parameters are frequently used to compare the performance of OVCs:

- Power conversion efficiency (PCE): ratio between the maximum power output of the cell and the incoming light power under standard test conditions (STC). Its value ranges from 0 to 100.
  $$\mathrm{PCE}\left(\%\right)=\frac{P_{max}}{P_{in}}\cdot 100=\frac{V_{mp}\cdot J_{mp}}{P_{in}}\cdot 100$$
- Fill factor (FF): ratio of the device maximum power to the product of the short-circuit current and open-circuit voltage. It is an indication of the efficiency of a PVC, and its value ranges from 0 to 1. In fact, FF values exceeding 0.75 can be considered outstanding in OSCs.
  $$\mathrm{FF}=\frac{V_{mp}\cdot J_{mp}}{V_{oc}\cdot J_{sc}}$$

Herein, we first discuss the characteristics and synthesis methods of GRMs, indicating the advantages and disadvantages of the different approaches in terms of yield, scalability, and performance. Next, the recent progress of GRMs in OSCs is discussed and device performances are compared. GRMs can be used as electrodes, active layers, and transport layers, and the role of the carbon nanomaterial is discussed. Finally, up-to-date matters and potential trends for future investigation of GRMs-based materials in this field are presented. 

## 2. Graphene-Related Materials: Characteristics and Synthesis Methods

### 2.1. Graphene: Synthesis and Characteristics

Graphene is a carbon nanomaterial containing of a monolayer of sp^2^ carbon atoms settled in a two-dimensional honey-comb structure. It is regarded as the “mother” of other graphitic materials since other C-based nanomaterials with different dimensionality can be constructed based on this building block [10]. It has a very high electron mobility of 2.5 × 10^5^ cm^2^/(V·s) and high electrical conductivity [11]. It is supposed to be the lightest existing material and the stiffest, more than 300 times stronger than steel, with a tensile strength of about 120 GPa [12], and an elastic modulus close to 10^3^ GPa [13]. It displays exceptional room temperature thermal conductivity, in the order of 5000 W/(m·K) [14], higher than that of copper or any other known material. Graphene can act as both a metal and semiconductor. It can conduct electricity better than copper and simultaneously has conduction and valence bands as in semiconductor but with no band gap; therefore, it is known as a semi-metal [15]. In addition, it displays good optical transmittance, is biocompatible [16], has optimal electrochemical stability, and can show luminance by doping. The combination of these extraordinary characteristics makes graphene ideal for use in PV devices. However, some concerns have to be solved. For instance, its resistivity is higher than that of conventional materials currently used as electrodes such as ITO; hence, it is essential to improve the transport of electrons without affecting other properties.

**Figure 4:**
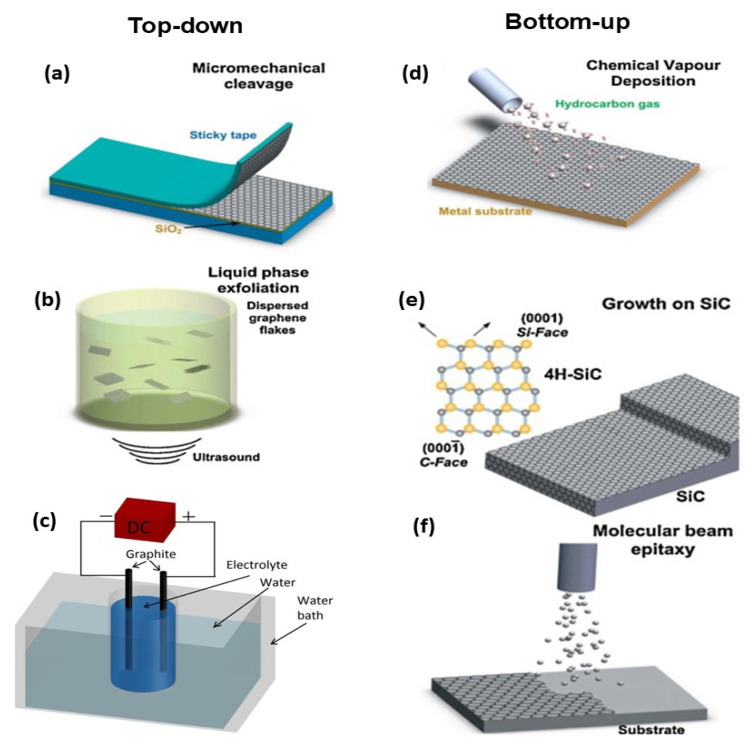
Illustration of the top-down and bottom-up methods for graphene production. (**a**) Micromechanical cleavage. (**b**) Liquid-phase exfoliation (**c**) Electrochemical exfoliation; (**d**) Chemical vapour deposition; (**e**) Growth on SiC; (**f**) Molecular beam epitaxy.

Graphene synthesis can be carried out following two main procedures, namely, bottom-up and top-down, as displayed in Figure 4. In the top-down technique, the initial material is graphite and the goal is to split it into graphene sheets, which can be performed in the solid or liquid state or in an electrochemical cell. The first approach to synthesize G was mechanical exfoliation, reported in 2004 by researchers at Manchester University [17], who obtained a monolayer via peeling a graphite surface with scotch tape. It produces high quality nanosheets and is inexpensive, but it is only useful at a laboratory scale since it yields very low quantities [17]. Liquid-phase exfoliation (LPE) directly disperses graphite flakes in a suitable solvent via solvothermal or sonochemical methods, thereby retaining the crystallinity of the graphene sheet. This approach is simple and scalable and is suitable for large mass production. Another type of exfoliation is electrochemical, which implies the use of ions that penetrate between graphite flakes for exfoliation. Depending on the applied voltage and the electrolyte, graphene with different properties can be obtained. This technique is inexpensive and environmentally friendly, being one of the most suitable for PV applications. 

On the other hand, the bottom-up approach [18] starts from smaller molecular precursors as units by techniques such as chemical vapor deposition (CVD), epitaxial growth or molecular beam epitaxy (Figure 4). CVD is a technique that yields G of very good quality using a substrate made of a transition metal, typically Cu, Pd, Ru or Ni [19]. CVD graphene is produced through two steps: (1) the charring decomposition of precursors that has to be carried out at elevated temperatures with the assistance of a metallic catalyst and on the surface of a substrate to inhibit the precipitation of soot during the gas phase. (2) The development of a graphene structure out of the dissociated C atoms that needs elevated temperatures as well. This method allows the synthesis of large graphene flakes, suitable for use in transparent electronics. The main drawbacks are that the metal catalysts can incorporate impurities in the graphene, the film thickness is difficult to tailor, and the substrate materials are fairly pricy. 

Epitaxial growth is typically performed on a semi-insulating SiC substrate that is graphitized via heating under vacuum conditions, leading to graphene formation at the outer surface of the substrate. This technique enables one to control the thickness by controlling the time and/or the temperature and is a promising route to synthesize high-quality large flakes with homogeneous thickness. One of its benefits is that the graphene layer is not transferred to another substrate, thus enabling the direct manufacturing of electronic devices [20]. Both semiconducting (p-and n-type) as well as semi-insulating SiC substrates are available for this approach, which allows diverse device architectures. The elevated temperature of the process guarantees the formation of well-ordered and spotless graphene. 

#### 2.1.1. Graphene Films and Crystals

**Figure 5:**
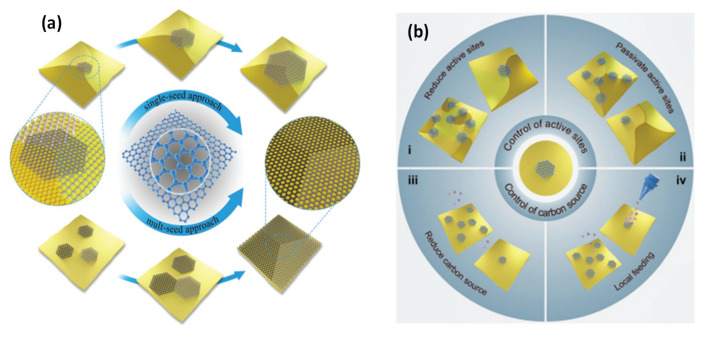
Approaches for the controlled growth of single-crystal graphene: (**a**) single-seed and multi-seed approaches. (**b**) Strategies for large single-crystal graphene via the single-seed approach: reduction of active sites, passivation of active sites, reduction of the carbon source, and local feeding. Reprinted from ref. [22], with permission from Wiley.

Many efforts have been devoted to grow monolayer graphene samples with large domains and reduced grain boundaries. Following the first synthesis of a graphene single crystal by Ruoff et al. [21], different crystal sizes have been effectively synthesized on Cu foil. Grain boundaries created during material synthesis influence both the intrinsic properties of materials and their applications. This effect is frequently found in graphene films developed via CVD. The chief approaches for increasing the graphene domain size and dropping grain boundaries are categorized into single-seed and multiseed methods, as depicted in Figure 5 [22]. In the single-seed approach, which involves growth from a single seed, control of the nucleation density is essential. The main strategies for reduced nucleation density fall into two types: one focuses on the control of active sites (either reduction or passivation) by modulation of surface characteristics of the metal substrates before graphene growth, and the other one mainly concerns the control of the carbon source supply (reduction of the carbon source or local feeding) during graphene growth. In contrast, in the multiseed approach, control of the grain orientation is critical to guarantee alignment of the graphene domains.

On the other hand, the CVD of C precursors on Cu substrates is the most popular method to produce large-area graphene films. To date, precise control of the number of graphene layers as well as the uniformity over a large area is still very challenging. Several parameters including gas partial pressures, ratio, and temperature are crucial. A high growth temperature and low carbon precursor concentration are beneficial for film growth. In addition, a pulsed-CVD approach can be used for film growth by exposing Cu to methane in an intermittent fashion during the growth steps. 

#### 2.1.2. Graphene Nanoribbons: Synthesis and Characteristics

**Figure 6:**
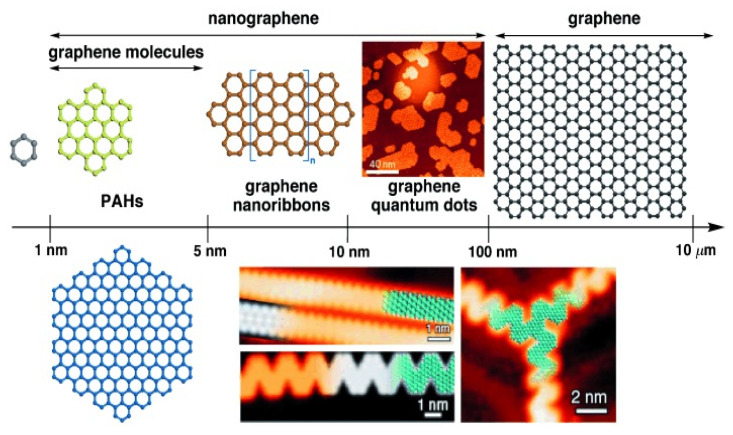
Depiction of graphene nomenclature depending on the size. G molecules are in the range of 1–5 nm; GNRs have a width smaller than 10 nm, and GQDs range from several to 100 nm. Reprinted from ref. [23], with permission from John Wiley & Sons, Inc.

Graphene nanoribbons (GNRs) are planar, finite, 1D materials with a graphitic lattice structure. They can be regarded as thin graphene stripes (narrower than 10 nm) but maintain a length-to-width ratio higher than 10 (Figure 6). They are classified according to the structure of their long edges, which can have an armchair, a zigzag or an intermediate character. This edge structure has deep implications for the electronic properties of the GNRs. Generally, GNRs are semiconducting, but the 1D band structure and specifically the size of the bandgap depend critically on the edge geometry. They have elevated electron mobility, a tunable bandgap, and adaptable electronic characteristics and are hence suitable for use in OSCs.

GNRs can be synthesized by top-down or bottom-up approaches. Top-down approaches include nanolithography patterning methods, unzipping of carbon nanotubes (CNTs) via an oxidative process or cutting of graphene or graphite precursors into thin graphene stripes via plasma etching [23]. However, these techniques lack precise control of the width and edge structure. To date, bottom-up fabrication by solution-mediated or surface-assisted procedures has been reported, which has several benefits in terms of atomically accurate GNR fabrication, and the success of this route relies on the proper design of appropriate reactants. Solution-mediated synthesis of GNRs is an efficient way to fabricate edge-controlled GNRs, such as heteroatom-terminated GNRs and alkyl chain installed GNRs. They are synthesized on metallic substrates such as silver, copper or gold, under high vacuum conditions, or in solution, via a two-step process of polymerization followed by cyclodehydrogenation from molecular precursors [24]. On the other hand, surface-assisted synthesis routes include radical-polymerization and surface-assisted cyclodehydrogenation.

#### 2.1.3. Graphene Quantum Dots

Graphene quantum dots (GQDs) are 0D carbon-based nanomaterials comprising one or a few graphene layers with sizes ranging from several to 100 nm (Figure 6). GQDs combine the excellent properties derived from graphene with the physicochemical features of the QDs such as the presence of a band–gap, edge, and quantum confinement effects and are hence highly suitable for energy applications and the electronics industry. They are non-toxic, biocompatible, solution processable, and show excellent photostability and tunable fluorescence.

The synthesis methods of GQDs can also be divided into top–down and bottom–up approaches [25]. In the top–down approach, the pristine nanomaterial (i.e., graphene, CNTs, carbon black) is cleaved by means of a heating/chemical treatment, ultrasonication or electrochemical exfoliation, leading to GQDs in the nanometer range. Although these strategies are appropriate for mass production given their simplicity and the abundant precursor materials, the indiscriminatory chemical cleavage prevents a fine control over the product size and morphology. On the other hand, the bottom–up approaches are based on the progressive development via carbonization, pyrolysis or CVD of tiny organic molecules (i.e., amino acids, salts, ascorbic acid, citric acid, glucose, etc.) in GQDs, enabling better control over the dimensions and fewer defects. 

### 2.2. Graphene Oxide: Fabrication and Characteristics

Graphene oxide (GO) is an oxidated form of graphene, and therefore its properties (which depend on the level of oxidation) are different from those of pristine graphene. GO nanosheets are amphiphilic and can be dispersed in aqueous solutions over a long period, displaying aqueous processability, which is critical for large-scale uses. They also form stable dispersions in many common organic solvents, which allows making films on numerous substrates via a simple drop-casting method, making it a suitable candidate for PV cells. Nonetheless, this nanomaterial behaves such as a wide-band–gap insulator due to sp^3^ hybridization of some carbon atoms. Thus, it has much lower electron mobility, and its electrical conductivity is typically lower than 10^−10^ S cm^−1^. It also displays lower mechanical strength and thermal stability, albeit the presence of oxygenated groups opens the way for a wide range of covalent and non-covalent functionalizations [26]. In particular, activation by N,N′-Dicyclohexylcarbodiimide (DCC) or thionyl chloride (SOCl_2_) and subsequent addition of amines, hydroxyls or isocyanates enables the covalent attachment of various functional groups to GO sheets [27]. On the other hand, GO films display high optical transparency and are non-toxic and biocompatible [28].

GO can be synthesized from graphite by means of different methods [26], namely, Staudenmaier, Hofmann, Brodie, and Hummers. Graphite oxide was initially synthesized by Brodie using KClO_3_ and HNO_3_. Later, Staudenmaier and Hofmann used concentrated H_2_SO_4_, HNO_3_, and KClO_3_ to make highly oxidized graphite. However, these methods were time-consuming, and unsafe since toxic gases (ClO_2_ and NOx) evolved. Later, Hummers developed an innovative route by substituting HNO_3_ and KClO_3_ with NaNO_3_ and KMnO_4_, and this has been the most widely employed method since the first exfoliation of graphene in 2004. Numerous investigators have tried to enhance Hummers’ method to make it more effective and/or sustainable. Some methods prevent the evolution of toxic gases by substituting NaNO_3_ by other oxidizing agents [29]. Another pathway to yield GO is the ultrasonication of graphite oxide in different solvents, an efficient means of attaining complete exfoliation, while it can severely damage the graphene sheets, even yielding graphene nanoplatelets (GNPs). High-yield sustainable procedures at a large-scale with natural oxidants such as citric acid have also been reported [30], and this prevented the development of toxic gases, being suitable for use in energy storage applications.

### 2.3. Reduced Graphene Oxide: Fabrication and Properties

Reduced graphene oxide (rGO) is derived from GO by chemical, thermal, and other methods in order to lower the oxygen content. The aim is to achieve nanomaterials analogous to pristine graphene. Nevertheless, defects and remaining functional groups markedly change the arrangement of the hexagonal lattice; consequently, the characteristics of rGO are intermediate between those of graphene and GO. Thus, rGO has an electrical conductivity about 10^2^–10^3^ times lower than that of pristine graphene. 

The approaches of converting GO into graphene can be classified into three types [31]: chemical, physical, and mixed, in which both types of factors come into play. In the physical ones, GO is heated at a high temperature (400–1200 °C) directly or via irradiation under a vacuum, inert or reducing atmosphere. The main benefit of this method is concurrent elimination of oxygenated moieties and reestablishing the graphene structure via sp^3^-sp^2^ rehybridization of C atoms during the thermal annealing. The main disadvantage is the difficulty to use it in aqueous medium or for layers grown on substrates; therefore, it is not appropriate for OSCs. Other routes are microwave radiation or reduction with an arc-discharge lamp or a pulsed laser [32], which leads to highly conductive rGO layers, enabling direct production of electronic devices.

The chemical route relies on the reaction of different chemicals with GO and can be performed uner ambient conditions or with mild heating. The chemical most widely employed is hydrazine (N_2_H_4_) [33]. However, its toxicity and the chance of integrating N into the structure make it unsuitable for production at an industrial level. Other frequently used reagents are Fe, hydroiodic acid (HI), sodium borohydride (NaBH_4_), hydroquinone, and hexamethylenetetramine [34]. The degree of reduction attained through chemical approaches can be improved via application of a solvothermal process that combines heating with chemical reactions under supercritical conditions [35]. This method enables one to synthesize N-doped rGO [36] with higher electric conductivity and simultaneously incorporates carboxyl groups in the hole boundaries, which is suitable for sensor applications. 

## 3. GRMs in Organic Solar Cells

GRMs have been applied in different parts of OSCs, mainly as transparent electrodes, hole/electron transport materials, and in active layers. The number of literature articles on OSCs containing GRMs is huge. The following sections summarize some of the most illustrative examples dealing with this topic. 

### 3.1. GRMs as Transparent Conductive Electrodes

A prerequisite for front electrodes (the one exposed to the light) of OSCs is to have elevated conductivity and high transparency. In this regard, the most common electrodes are indium and fluorine doped tin oxide (ITO and FTO). ITO has more than 80% transmittance in the visible range and about 0.1 S cm^−1^ conductivity on a glass substrate [33], but it presents several drawbacks, i.e., sensitivity to basic and acidic media, brittleness, high cost because of a lack of sources of indium, and difficulty in patterning. Besides, processing at high temperatures is required to manufacture ITO and FTO with good performance. Thus, novel materials such as CNTs, metal nanoparticles, metal oxides, and recently GRMs have been explored as substitutes for ITO [37,38]. In particular, GRMs offer high conductivity, elevated transmittance in the visible region, outstanding stiffness, and durability combined with high flexibility and potential low cost. The transmittance of GRM thin films is the principal parameter accounting for their properties and it is inversely proportional to their conductivity and thickness. In this regard, CVD graphene provides high-quality films with better conductivity and transparency than those synthesized via Hummer’s method. Besides, electrodes incorporating GO have higher transmittance than CVD graphene. In addition, graphene comprised of a few layers is preferred over monolayer graphene due to its lower sheet resistance and air permeability [39]. Overall, a transparent conductive electrode requires a good balance between transparency and conductivity. The device performance and PV parameters of OSCs incorporating GRM-based electrodes are summarized in Table 1.

**Figure 7:**
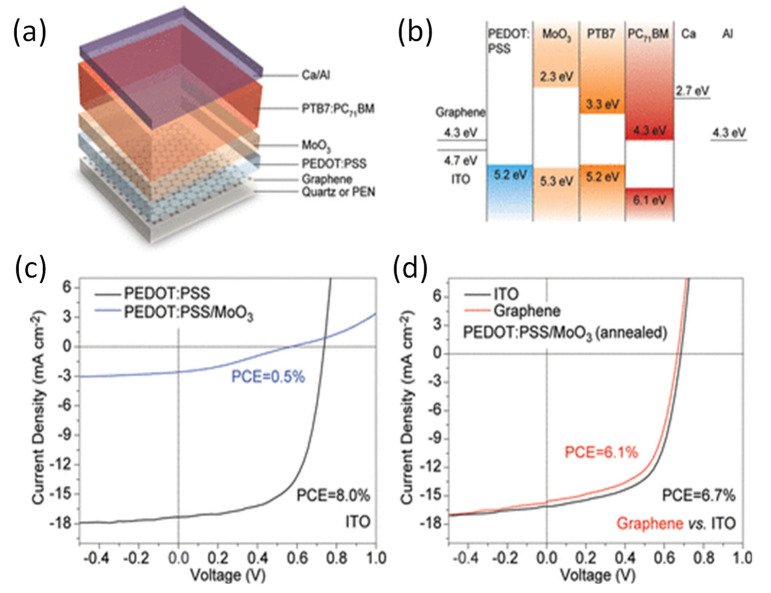
(**a**) Cell structure, (**b**) energy levels, and (**c**,**d**) *J-V* curves for graphene- and ITO-based OSCs. Taken from ref. [45], with permission from the American Chemical Society.

The comparison of the PCEs obtained for the different cell configurations corroborates that CVD graphene has better performance than GO, rGO or EG due to its higher quality and better conductivity. Thus, the highest PCEs (6.1 and 7.1%) were reported for OSCs with CVD-graphene as the anode [45]. The device structure and the energy levels are displayed in Figure 7. The active layer comprised a blend of a semiconducting polymer donor (PTB7) and a fullerene acceptor (PC_71_BM). The hole transport layer was PEDOT:PSS, which was modified with isopropyl alcohol to guarantee homogeneous coating on the graphene electrode. An additional layer of MoO_3_ was deposited onto the graphene/PEDOT:PSS in order to block electrons and prevent charge recombination at the interface between the polymer and the nanomaterial. This graphene-based OSC showed comparable performance to that of an equivalent ITO-based cell under identical conditions (Figure 7d). These G-based devices showed exceptional durability and did not lose performance after one hundred bending cycles.

**Figure 8:**
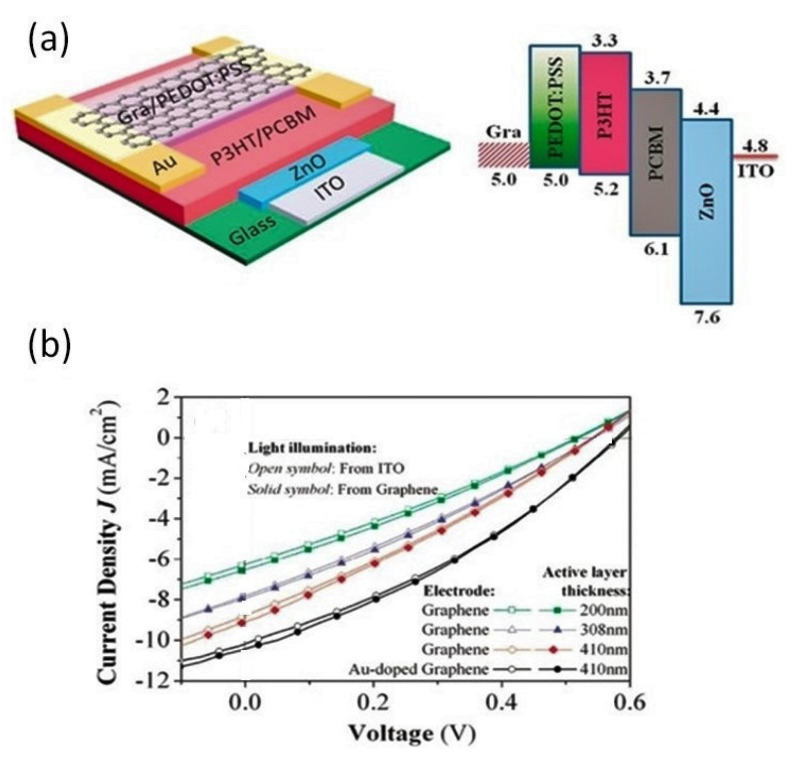
(**a**) Representation of an OSC with an ITO/ZnO/P3HT:PC_61_BM/PEDOT:PSS + Au/CVD-G (1L) structure; (**b**) *J–V* curves measured from both sides of the OSC for different thicknesses of the active layer. Reproduced from ref. [42], with permission from the American Chemical Society.

Incorporation of foreign atoms/molecules into graphene is a worthy path to increase its conductivity and simultaneously control the work function of the modified graphene. For instance, the doping of graphene with bromine under an inert atmosphere led to transparent electrodes with high stability and a nearly 5-fold increase in conductivity with only 2–3% loss of transmittance. This was ascribed to the encapsulation of bromine by intercalation between the graphene sheets. Semitransparent OSCs with a blend of poly(3-hexylthiophene) (P3HT):PC_61_BM as an active layer, CVD-single-layer graphene doped with Au nanoparticles, PEDOT:PSS as the top electrode, and ITO as the bottom electrode have also been manufactured, as depicted in Figure 8 [42]. The doping resulted in about a 390% increase in conductivity in comparison to the cell with raw graphene, and a PCE of 2.70% was reached once irradiated from the graphene side, ascribed to an improvement in transmittance. Improved performance was also found upon increasing the thickness of the active layer. Higher efficiency could be expected by optimizing the composition and processing conditions.

The performance of OSCs incorporating the P3HT:PCBM active layer is restricted due to the little difference in energy between the LUMO of PCBM and the HOMO of P3HT that yields a low *V_oc_* of about 0.59 V. In order to attain better cell properties, novel materials such as indene-C60 bisadduct (ICBA) with a higher LUMO energy level have been employed. Thus, the cell with P3HT:ICBA as an active layer, graphene doped with bromine, PEDOT:PSS as the upper electrode, and Al as the bottom electrode attained a *V_oc_* of 0.76 V, albeit only 2.10% PCE was reached [53]. This could be due to a poor charge transfer throughout the cell interfaces, one of the major reasons accounting for the low performance of OSCs. Other factors including the presence of impurities, oxygen doping, charge dipole formation, and phase segregation can also be responsible for the observed behavior. 

Exfoliated graphene synthesized via electrochemical exfoliation of graphite was deposited onto a polymeric substrate, poly(ethylene 2,6-naphthalate) (PEN), and the mixture was used as the anode in OSCs. The cell comprising PTB7:PC_71_BM as the active layer led to an efficiency of 4.23% that was preserved upon application of hundreds of bending cycles. This EG-based electrode showed a low sheet resistance, high toughness, and a transparency of 70% combined with good solution processability Conversely, very low PCE values have been attained using rGO-based electrodes due to the presence of numerous defects, which significantly restrict the cell efficiency. Thus, thermally annealed thin rGO films (about 20 nm) with 65% transparency deposited onto a PET substrate by spin coating only led to a PCE of 0.78% [47] and *V_oc_* of 0.56 V. Remarkably, the cell obtained could resist more than 1000 cycles upon bending, whereas conventional ITO-based cells typically deteriorate and fail. 

Higher PCE (8.48%) has been reported for cells incorporating Au-doped GNRs as the anode [48]. Their improved performance is ascribed to the incorporation of WO_3_ as electron blocking and the optimization of the doping concentration. Thus, as this concentration increased up to 3 mM, the conductivity increased, but above 5 mM it started to decrease. These results suggest that by controlling the doping concentration, the conductivity values and hence the device performance can be optimized. Besides, the processing conditions and the thickness of the ETL, HTL, as well as the active layer were optimized to prevent exciton recombination and the probability of losing charge at the anode and cathode.

The encapsulation of a silver nanowire (Ag NW)-based electrode with graphene is another method recently suggested to improve the durability of OSCs. Thus, AgNWs were covered on both sides with a GO layer using scalable and solution-compatible processes, resulting in a sandwich-like structure [49]. The GO layer protects the AgNWs from the acidity of the PEDOT:PSS hole transport layer and from degradation due to atmospheric sulfidation. Thus, a fivefold improvement in the cell durability was reported. However, the PCE was still low (2.6%); hence, device configuration should be optimized to attain better performance. Graphene electrodes doped with GQDs-Ag NWs were also fabricated and incorporated into flexible OSCs [57]. A sheet resistance of about 92 Ω/sq was reported, resulting in 3.66% efficient OSCs. In addition, a significant improvement of the bending flexibility of the electrode was achieved. Hybrid electrodes based on graphene-AgNWs were also deposited on PET substrates. The addition of graphene improved the electrical and optical characteristics, increasing the flexibility and durability. Inverted, 6.47% efficient OSCs were manufactured with a PTB7:PC_71_BM active layer on graphene-AgNWs [58]. The highest efficiency using Ag-NWs and graphene-based electrodes (13.4%) with *V_oc_* of 0.83 V, *J_sc_* of 23.20 mA cm^−2^, and FF of 69.8% was reported by Zhang and coworkers [59] via the addition of a thin PEDOT:PSS layer with high conductivity that modified the rough surface of graphene-AgNWs and prevented the divergence between the work function of the interfacial layer and the electrode. Besides, the device showed exceptional flexibility and it preserved about 85% of the original efficiency upon more than 1000 bending cycles. This work corroborates that GRMs-AgNW environmentally friendly electrodes are a very good alternative to substitute ITO.

**Figure 9:**
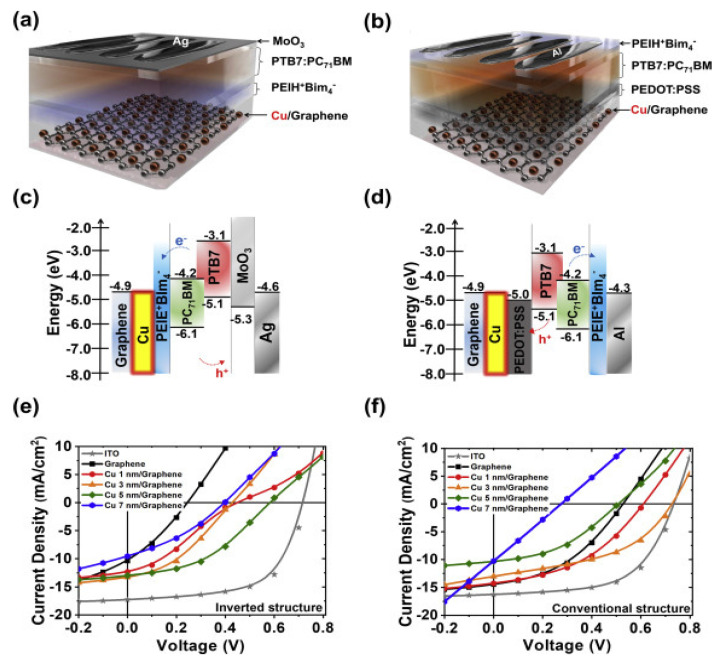
(**a**) Inverted and (**b**) conventional PTB7:PC_71_BM cells with graphene-Cu as bottom electrodes. (**c**,**d**) Representation of the energy levels of (**a**,**b**). *J–V* plots of the inverted and conventional OSCs (**e**,**f**). Taken from ref. [50], with permission from Elsevier.

Hybrid transparent electrodes based on Cu-grid and graphene film have also been developed as bottom electrodes in conventional and inverted OSCs [50]. The addition of the Cu layer improved the PCE by 1.3 and 5-fold for the two configurations, respectively, compared to the cells with just graphene (Table 1). The characteristics of the individual graphene and Cu layers are critical parameters to be improved with the aim of attaining good optical transmittance and electrical conductivity. Thus, it was found that increasing Cu thickness increases the conductivity (Figure 9) but reduces the transmittance due to the opacity of the Cu layer.

Films of Cu-based Al-doped ZnO (AZO) were prepared and incorporated as electrodes in OSCs with a structure of PEDOT:PSS/P3HT:PCBM/PCBM/Ca/Al. A graphene layer between the AZO and Cu layers was added that prevented the contact between Cu and oxygen during the preparation process of AZO, thus reducing the system transmittance. A conductivity of 2.5 × 10^4^ S cm^−1^, a transmittance in the VIS region higher than 82%, and a PCE of 4.63% were reported [60], yielding an improvement of 95% compared to ITO-based OSCs. Additional investigations on graphene/metal mixtures are desirable in order to attain better performances.

**Figure 10:**
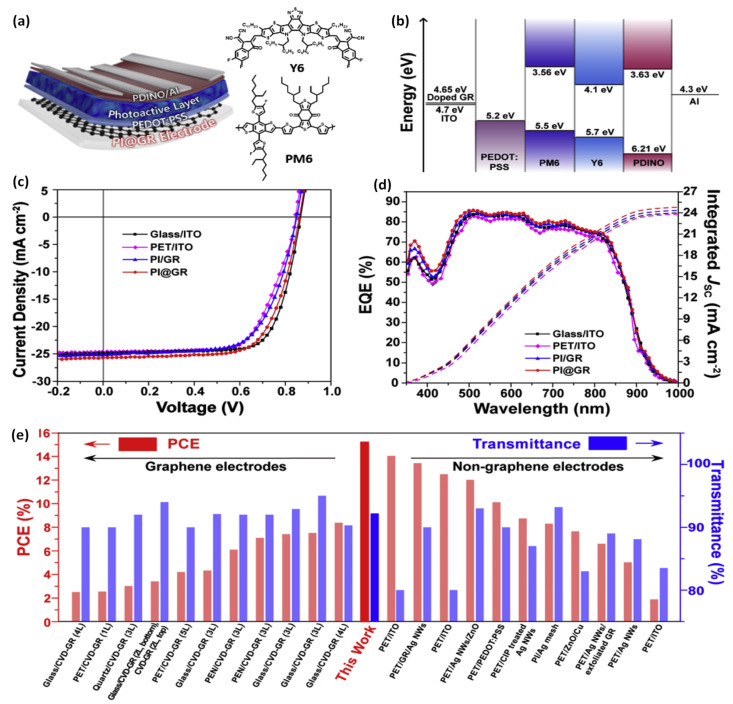
Characteristics of OSCs using a PI-graphene film electrode: (**a**) Schematic representation of the cell structure; (**b**) energy level diagram. (**c**) *J–V* plots, and (**d**) external quantum efficiency (EQE) spectra (solid line) with integrated current density (dashed line). (**e**) Comparison of transmittances and cell performances of OSCs. Taken from ref. [61], with permission from Elsevier.

The highest PCE for cells incorporating graphene-based electrodes (15.2%) has been recently reported by Koo and coworkers [61]. In such work, a highly flexible, thermally stable, and robust electrode was obtained via a combination of polyimide (PI) with a CVD-graphene film by a layer-by-layer assembly process (Figure 10). PI has a dual behavior as a transporter film for graphene and a substrate for the electrode. The hybrid electrode showed a very clean surface with a transmittance higher than 90% (transmittance over ITO above 350 nm), a conductivity higher than 1.2 × 10^3^ S cm^−1^, and high thermal stability. It also showed exceptional mechanical strength up to thousands of bending cycles with an efficiency comparable to that of an ITO-based reference device (15.7%). Larger cells were manufactured to investigate their scalability, although a slight worsening in the cell performance was found upon increasing the size of the device. The cell with an active area of 0.5 cm^2^ showed an efficiency of 12.7%, better than the PCE of a cell with similar configuration but incorporating ITO as the electrode, irrespective of its larger active area.

### 3.2. GRMs as Active Layers

As mentioned earlier, most OSCs are based on the BHJ architecture [7], which comprises a bicontinuous nanoscale network of a donor and acceptor that maximizes the interfacial area. The most widely used active layers in these kinds of cells are based on poly-3-hexylthiophene (P3HT) or poly-3-octylthiophene (P3OT) as the donor and PCBM or PC_70_BM as the acceptor. However, their efficiency does not exceed 5%. Hence, innovative materials for both the donor and acceptor that absorb light more efficiently, with improved HOMO/LUMO matching, more effective exciton dissociation, and enhanced charge mobility with good stability are sought. In this regard, GRMs show great potential due to their huge specific surface area and 2D structure at the nanoscale. In this section, the use of GRMs as acceptor materials is discussed, and the efficiency along with the cell configuration are summarized in Table 2.

In 2008, Liu and coworkers [62] were pioneers and described the use of solution-processable graphene modified with isocyanate as an acceptor in OSCs. The active layer was prepared by spin-coating a mixture of P3OT and functionalized graphene in 1,2-dichlorobenzene and was subsequently annealed at 160 °C for 20 min. The cell, with a configuration of ITO/PEDOT:PSS/P3OT:G/LIF/Al, displayed an efficiency of 1.4% for a graphene concentration of 5 wt%. The same researchers used 10 wt% of the modified graphene and P3HT as the donor in an analogous device [63], reaching a maximum efficiency of 1.1% upon annealing at the same temperature for 10 min. The enhancement compared with the cell without graphene was explained considering the quicker electron transport via the carbon nanomaterial. Besides, annealing has been reported as an effective means to improve PCE given that it significantly increases the cell *V_oc_*. A similar type of OSC has been developed by other authors [67,68,69,70], yielding quite low performances (0.6–1.5% PCE). For instance, the incorporation of a mixture of graphene and functionalized multiwalled carbon nanotubes (f-MWCNTs) as the acceptor and P3HT as the donor was attempted, but the resulting device had a lower PCE, given that graphene has larger surface area, improved ability as a charge carrier, and enhanced dispersibility compared with the f-MWCNTs. 

**Figure 11:**
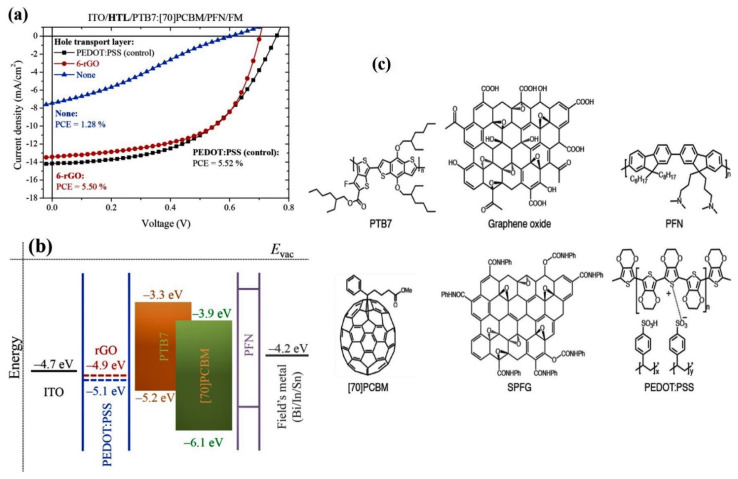
(**a**) *J–V* plots of the ITO/PEDOT:PSS/PTB7:PCBM:SPFG/PFN/FM cell; (**b**) energy level diagrams of the device; (**c**) Chemical structures of the compounds. Reproduced from ref. [78], with permission from Elsevier.

Solution-processable functionalized graphene (SPFG) has been integrated as an additional constituent in a PTB7:PCBM active layer, leading to a cell with structure of ITO/PEDOT:PSS/PTB7:PCBM:SPFG/PFN/FM (Figure 11 [78]). Different amounts of SPFG were tested, and the optimum concentration was 4 wt%. An increase in PCE of 22% was measured in relation to reference cells without SPFG. The improvement was attributed to additional charge-carrier pathways, which lead to better charge transport and collection, hence an increase in *J_sc_*. At higher concentrations, the PCE decreased due to graphene agglomeration.

Polymer grafting is an alternative strategy employed to improve the PCE of OSCs. CH_2_OH-terminated P3HT was grafted onto the COOH groups of GO via an esterification reaction [69], leading to a P3HT-g-GO derivative soluble in many organic solvents, which is beneficial from a processability viewpoint. The chemical grafting can improve light absorption and electron mobility. However, the performance of the cell, with the structure of ITO/PEDOT:PSS/G-g-P3HT:C60/Al was only 0.61%. The same group [70] grafted C_60_ fullerene to rGO via a lithiation reaction, and the grafted samples acted as electron acceptors in the active layer of the cell: ITO/PEDOT:PSS/C60-g-rGO:P3HT/Al. Grafting led to a better electron transport pathway, but the PCE was only 1.22%.

**Figure 12:**
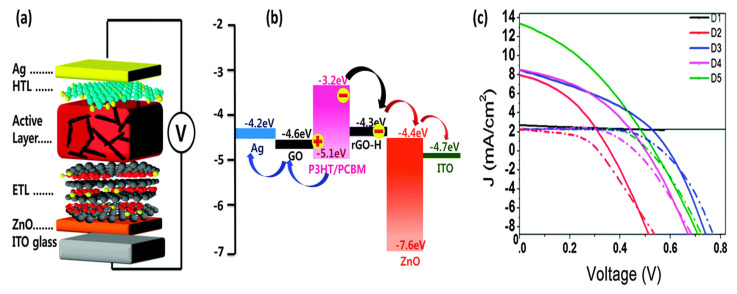
(**a**) Structure of the cell with GO as the HTL and PCA-rGO as the ETL and in the active layer, (**b**) energy levels, and (**c**) *J–V* curves of D1 to D5 cells (solid lines) and the corresponding dark currents (dashed lines). D1: ITO/ZnO/P3HT/PEDOT:PSS/Ag; D2: ITO/ZnO/P3HT:rGO/PEDOT:PSS/Ag D3: ITO/ZnO/P3HT:PCA-g-rGO/PEDOT:PSS/Ag; D4: ITO/ZnO/P3HT:PCA-rGO/GO/Ag; D5: ITO/ZnO/PCA-g-rGO/P3HT:PCA-g-rGO/GO/Ag. Reproduced from ref. [64], with permission from the Royal Society of Chemistry.

Another method consisted of the covalent grafting of a polyaromatic derivative with amphiphilic characteristics, 1-pyrenecarboxylic acid (PCA), onto GO followed by hydrazine reduction to improve its processability and optical transmittance without sacrificing the conductivity [64]. Thus, PCA-g-rGO had a high conductivity close to 1.5 × 10^3^ S cm^−1^, about a 4-fold increase compared to that of pristine GO. The PCA-g-rGO dispersion was stable for a few weeks. The hybrid cell, including the rGO-PCA in the active and electron transport layers, with an structure of ITO/ZnO/PCA-g-rGO/P3HT:PCA-g-rGO/GO/Ag (Figure 12), showed a PCE of 2.85%, about 16 times higher than that of devices with only P3HT (0.18%), and about 2.4 times higher than that of the cell with only PCA-g-rGO in the active layer, attributed to the increase in the *J_sc_*.

Non-covalent functionalization has also been explored as an easy alternative to improve PCE. Thus, Lyu and coworkers [80] developed a novel functionalized GO with PTB7, a conjugated polymer, by simple ultrasonication in ethanol and evaporation in an oil bath. The PTB7 chains adhere to the monolayered dispersed GO sheets via π–π stacking. The resulting P-GO was incorporated in different concentrations into the PTB7:PC71BM active layer. The electrical parameters remained almost unchanged with increasing active layer thickness, and 1 wt% P-GO was found to be the optimal concentration, resulting in a high efficiency (7.52%).

**Figure 13:**
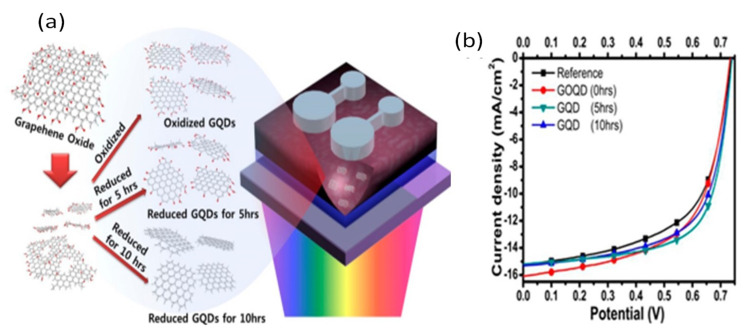
(**a**) Depiction of the chemical routes for the preparation of GOQDs and rGOQDs from GO; (**b**) *J–V* plots of cell with different kinds of GQDs. Reproduced from ref. [74], with permission from the American Chemical Society.

GNRs, which are highly soluble and easily processable into thin films, have also been tested as electron acceptors in OSCs with PTB7-Th as the electron donor [65]. The mixtures have broad and intense absorption from 350 to 800 nm, leading to a PCE of 7.6%, comparable with the best PTB7-Th-based nonfullerene solar cells, since each ribbon can accept large numbers of electrons. On the other hand, 0D GQDs constitute a suitable route to tailor the band gap of graphene. GQDs with sizes in the range of 3–5 nm have been used as acceptors and P3HT as a donor in different cell devices [71,72,73,74]. Moderate efficiency (5.24%) was found for those obtained from double-walled CNTs synthesized via solution processing [73]. Those electrochemically synthesized [71] led to an efficiency of 1.28%, while GQDs synthesized by a hydrothermal technique and functionalized with aniline, in a cell with P3HT as donor, resulted in a PCE of 1.14%. Nevertheless, among the GQDs, the highest PCE (7.6%) was found for partially reduced GQDs, ascribed to an optimal balance of absorptivity and conductivity (Figure 13 [74]). Graphene oxide quantum dots (GOQDS) showed enhanced optical absorptivity due to the numerous oxygenated functional groups but showed increased *J_sc_* with reduced conductivity. On the other hand, reduced GQDs had improved FF, attributed to the improved carrier conduction. Therefore, an intermediate degree of reduction of the GQDs is desired. 

**Figure 14:**
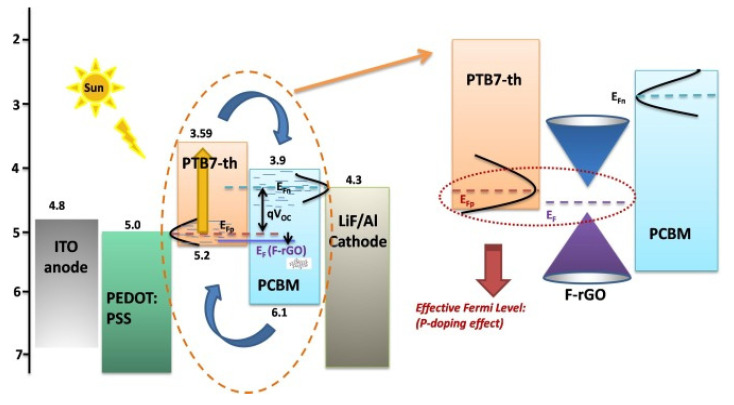
Energy levels of the cell ITO/PEDOT:PSS/PTB7-th:PCBM:F-rGO/LiF/Al, showing the p-doping effect. Taken from ref. [81], with permission from Elsevier.

The doping of graphene has also been tested in order to modulate the band–gap structure. Thus, N-doped graphene blended with P3HT:PCBM as an active layer provided transport paths specific for certain charge carriers, resulting in a moderate PCE (4.48%) [75]. In another study, reduced-fluorinated graphene oxide (F-rGO) was studied as an additional component in the active layer PTB7-th:PCBM. The addition of F-rGO improved charge generation and transport and led to a more efficient exciton dissociation (Figure 14), attributed to a p-type doping. Thus, cells with 5 wt% F-rGO attained an efficiency of 7.36%. 

### 3.3. GRMs as the Hole-Transport Layer

In order to attain effective OSCs, the work functions of the anode and the cathode should match the HOMO of the donor and the LUMO of the acceptor, respectively, to reduce electron- and hole-extraction barriers. In addition, these barriers can be decreased by placing a hole-transport layer (HTL) within the anode and the active layer, as well as an electron-transport layer (ETL) in the middle of the cathode and the active layer. The main roles of these layers are as follows [9]: (a) reduce the barrier for charge extraction; (b) extract only one type of charge carrier; (c) adjust the active layer morphology; (d); enhance the electron-active layer interfacial stability; (e) act as an optical spacer. GRMs have been employed in both the HTL and ETL due to their tunable energy levels that allow improved charge transport, solution processability, proper work function, potential low cost, and reduced corrosion for the ITO electrode. The use of GRMs as the HTL is discussed in this section, the acceptor materials are described, and the PCE along with the cell configuration is summarized in Table 3.

**Figure 15:**
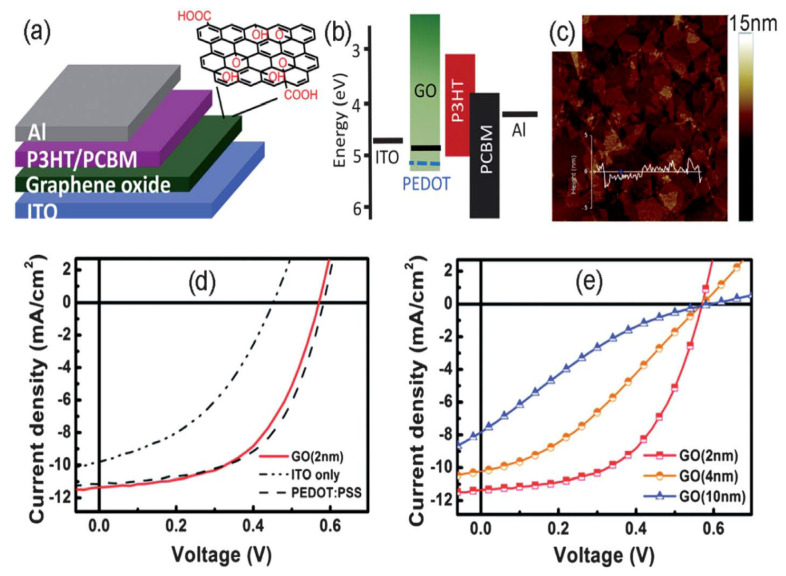
(**a**) Representation of the OSC with GO as the HTL. (**b**) Energy levels of the device (**c**) AFM micrograph of a 2-nm-thick GO film. (**d**) *J–V* curves of the cells without HTL and with PEDOT:PSS and GO as the HTL. (**e***) J–V* plots of the ITO/GO/P3HT:PCBM/Al devices. Taken from ref. [87], with permission from the American Chemical Society.

The work function of GO is about −4.8 eV that matches P3HT as a donor for effective hole extraction (Figure 15). In 2010, Li and coworkers [87] described the application of GO as the HTL in OSCs for the first time. GO was homogeneously deposited onto an ITO anode by simple spin coating in aqueous solution. The developed cell, ITO/GO/P3HT:PCBM/Al, showed an FF of 54% and a PCE of 3.5%, values considerably better than those of the cell without HTL and comparable to the device incorporating PEDOT:PSS (3.6% PCE). The FF and PCE decreased as the thickness of the GO layer increased, attributed to the increased resistance due to the insulating nature of GO. A similar approach was reported by Gao et al. [86] for an inverted device configuration. A uniform GO layer was deposited on the P3HT:PCBM active layer via spin coating its solution in anhydrous butyl alcohol. The inverted OSC device with an optimal GO layer thickness of 2–3 nm showed a V_OC_ of 0.64, FF of 64%, and PCE of 3.60%. These authors reported that GO could dope P3HT at the surface of the active layer because of its large number of oxygenated groups with high H^+^ concentration. The doped P3HT thin layer enabled a good Ohmic contact between the active layer and the top metal electrode. Nonetheless, to attain better performance, the conductivity of the GO acting as the HTL should be improved. 

**Figure 16:**
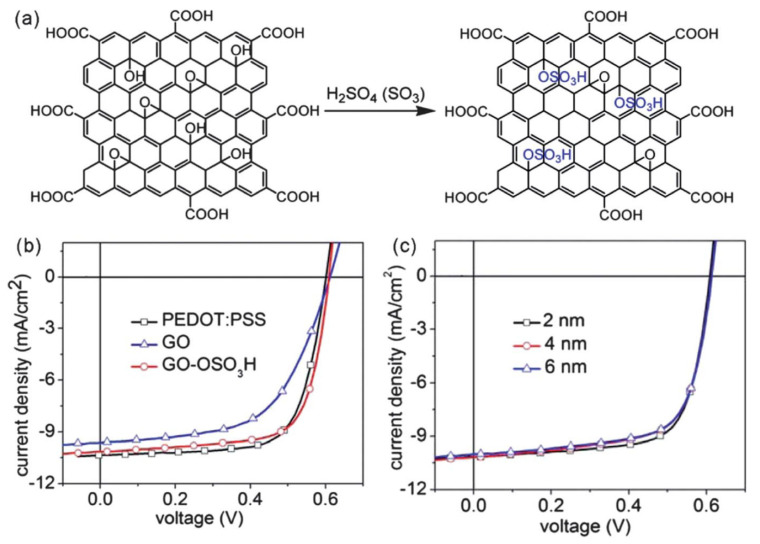
(**a**) Chemical structure and path for GO-OSO_3_H synthesis. (**b**) *J–V* plots of the OSCs with PEDOT:PSS (25 nm), GO (2 nm), or GOOSO_3_H (2 nm) as the HTL. (**c**) *J–V* plots of the OSCs with GO-OSO_3_H as the HTL, for different layer thicknesses. Reproduced from ref. [93] with permission from the American Chemical Society.

Sulfated graphene oxide (GO-OSO_3_H) prepared by functionalizing GO with H_2_SO_4_ has also been used as the HTL in similar P3HT:PCBM-based cells (Figure 16) [93]. The device showed a PCE of 4.37%, one of the highest described for OSCs comprising this active layer. Besides, the device properties were hardly dependent on the thickness of the GO-OSO_3_H, in contrast to the above-mentioned cells with the insulating GO as the HTL.

Blending with polymers is another path to improve device performance. For instance, a GO/PEDOT:PSS composite was incorporated as an HTL in a device with PCDTBT:PC_71_BM as the active layer. Blending GO and PEDOT:PSS in aqueous medium significantly increased the viscosity of the solution, making a uniform film that could be directly deposited onto ITO by an adhesive lamination procedure. The resulting cell had an efficiency of 4.28% [83], much better than that of cells comprising PEDOT:PSS or GO as the HTL that had PCEs of 3.56 and 2.77 and 3.57%, respectively. Besides, they showed better stability and reproducibility. The enhanced properties were attributed to the good matching between the work functions of PEDOT:PSS and GO, which decreased resistance and enhanced charge mobility. In addition, GO acted as an electron blocker, leading to an increased shunt resistance. 

Metal nanostructures are often incorporated in OSCs to expand their light-harvesting capability. These nanostructures can be incorporated at the active layer–electrode interfaces. In this regard, a novel amphiphilic nanocomposite (Au@PEG–GO) comprising gold nanoparticles (Au NPs), GO, and poly(ethylene glycol) (PEG) to improve solubility was developed [84], and it had good dispersibility in aqueous solution and different organic solvents. The deposition of Au NPs on GO sheets ensured a homogeneous nanoparticle dispersion, thus preventing aggregation in the physical mixture. The Au@PEG–GO acted as the HTL in the cell: ITO/PEDOT:PSS(Au@PEG–GO)/PBDTTT-CT:PC71BM/Ca/Al, which showed a good efficiency (7.26%).

In order to enhance the electrical conductivity, rGO has also been tested as the HTL. Thus, a solution-processable reduced graphene oxide (spr-GO) was developed via reduction with a hydrazine derivative in aqueous medium [92]. For comparison, r-GO synthesized via conventional hydrazine reduction was also prepared. Both pr-GO and r-GO displayed nearly five times higher conductivity than pristine GO. spr-GO was homogenously deposited onto ITO via solution-casting, while rGO formed large agglomerates. The OSC with a configuration of ITO/spr-GO/P3HT:PCBM/Al showed a PCE of 3.63% and an FF of 66.7%. Besides, the cell incorporating spr-GO had a longer lifetime than that of a reference with PEDOT:PSS as HLT. Another strategy to improve electrical conductivity is polymer grafting. In this regard, a polyacrylonitrile-grafted rGO nanocomposite (PRGO) was manufactured via in situ polymerization of a functionalized GO in the presence of acrylonitrile [85]. It showed uniform morphology, fairly high electrical conductivity (0.9 S cm^−1^), high work function (4.9 eV), and outstanding environmental stability. PRGO was incorporated as the HTL in a cell with configuration of ITO/PRGO/PEDOT:PSS/PTB7-th:PBCM/Al, which exhibited an efficiency of 7.24% and an FF of 64%, values similar to those of a reference device with PEDOT:PSS (PCE of 7.17% and FF of 66%). Besides, the cell based on PRGO displayed higher durability.

**Figure 17:**
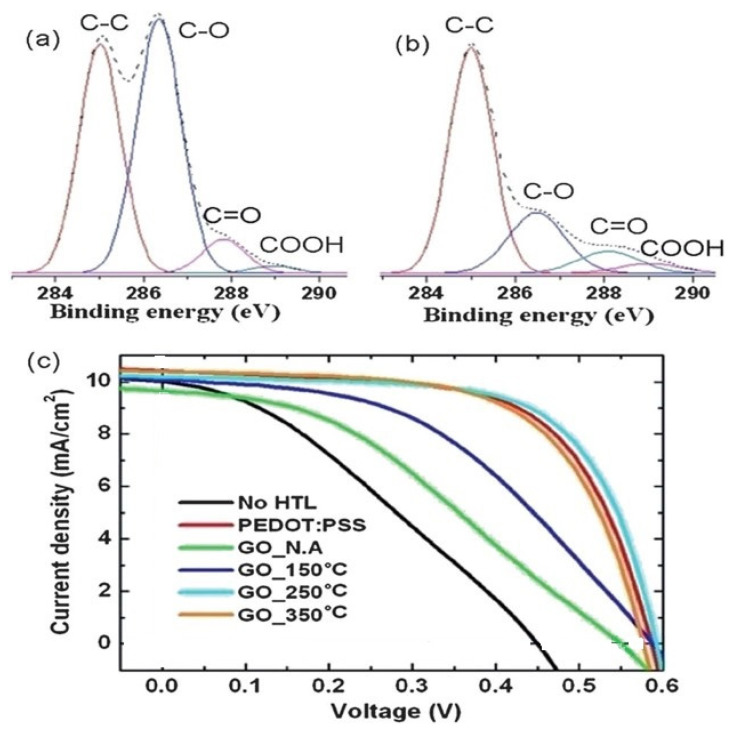
XPS spectra of GO (**a**) without thermal treatment and (**b**) with thermal annealing at 250 °C. (**c**) *J–V* curves of OSCs with thermally annealed GO as the HTL. Reproduced from ref. [94], with permission from Elsevier.

In addition to the above-mentioned methods, thermal annealing of GO has been shown to be demonstrated to be effective to attain highly conductive rGO. Throughout thermal annealing, oxygenated groups are decomposed and detached as gas molecules. Nevertheless, this method is restricted to OSCs on glass substrates. OSCs with thermally annealed GO for 10 min in air at 150, 250, and 350 ºC have been developed (Figure 17 [94]). The conductivity of GO significantly increased upon the thermal treatment. Accordingly, the cell efficiency increased from 1.5 to 4.0%, value similar to that of the reference cell with PEDOT:PSS as the HTL. Furthermore, the cell with annealed GO displayed improved environmental stability. 

The potential of conductive nanofillers to enhance the conductivity of GO as the HTL has also been examined. In particular, single-walled carbon nanotubes (SWCNTs) have been mixed with GO via sonication in aqueous solution combined with spin coating to produce a homogeneous GO:SWCNT nanocomposite. OSCs with P3HT:PCBM as the active layer showed an efficiency of 4.1%. The same nanocomposite was subsequently used as the connection layer for building series of tandem OSCs in order to improve light absorption.

**Figure 18:**
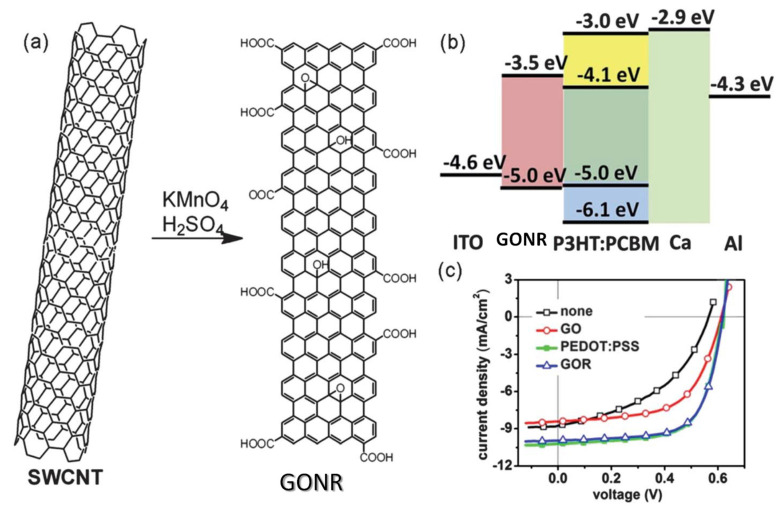
(**a**) Representation of the synthesis of graphene oxide ribbon (GONR) by oxidative unzipping of SWCNTs. (**b**) Energy levels of the GOR-based OSC cell. (**c**) *J–V* plots of the OSCs without and with GO, PEDOT:PSS or GONR as the HTL. Reproduced from ref. [91], with permission from John Wiley and Sons.

Graphene oxide nanoribbons (GONR) synthesized via oxidative unzipping of SWCNTs combined with an additional oxidation stage were fabricated and used as the HTL [91]. The GONR combined the aqueous processability of GO and the semiconducting character of GNRs. The GONRs had a HOMO of −5.0 eV and LUMO of −3.5 eV that promoted hole transport and electron blocking, hence reducing electron–hole recombination at the anode (Figure 18). The OSC showed analogous performance to that of the cell with PEDOT:PSS (4.14% PCE) and significantly improved that of an equivalent cell with GO. Moreover, the device showed better stability.

The doping of rGO with heteroatoms is another strategy that can result in improved performance. Thus, GO was simultaneously functionalized and reduced with a hydrazine derivative incorporating fluorine atoms, and the resulting fluorinated-reduced graphene oxide (F-rGO) was deposited via spin-casting. Due to the high electronegativity of the doping atoms, F-rGO has a work function of 5.1 eV and was used as the HTL in an OSC with PTB7:PC_71_BM as the active layer and the field’s metal (Bi/In/Sn: 32.5/51/16.5 wt%) as the top electrode, leading to a PCE of 5.73% [96]. An improved PCE of 7.67% was obtained when F-GO was used as interlayer between ITO and PEDOT:PSS. A similar cell based on F-GO was prepared by other authors [97], using a hydrazine derivative with seven fluorine atoms. The resulting F-rGO was well dispersed in organic solvents without the need for surfactants, resulting in good film-forming ability and high conductivity. The cell with F-rGO as the HTL exhibited higher PCE (8.6%) and durability than cells with GO HTLs, even higher than those of reference cells incorporating PEDOT/PSS. These observations confirm that F-rGO has great potential as the HTL material to substitute traditional PEDOT/PSS at a lower cost. The same approach was used in cells based on conjugated polymer (PBDB-T) and non-fullerene acceptor (ITIC) as the active layer, leading to a PCE of 5.3% [98]. The inhomogeneity of the F-rGO layer could be the factor limiting the device performance. 

### 3.4. GRMs as the Electron-Transport Layer

The work function of electron transport layer (ETL) materials needs to be low enough to match the LUMO of the acceptor in the active layer; this promotes electron extraction and subsequent mobility, thus minimizing series resistance of the OSCs. GRMs with tailorable energy levels via chemical modification can be employed as the ETL too, as summarized in Table 4.

In 2012, Liu and coworkers [101] used cesium-neutralized graphene oxide (GOCs) as the ETL for OSCs. The GO-Cs showed a work function close to 4.0 eV, that matches very well with the LUMO of the PCBM acceptor. The normal device, with structure: ITO/GO/P3HT:PCBM/GO-Cs/Al, showed an efficiency of 3.67%. The inverted device (ITO/GO-Cs/P3HT:PCBM/GO/Al) had a PCE of 2.97%. Other authors [102] prepared a pyrene-functionalized fullerene derivative that was non-covalently anchored to r-GO via π-π interactions between the pyrene aromatic rings and the r-GO ones. OSC with this r-GO/fullerene composite as the ETL showed an efficiency of 3.9%, superior than that of the reference cell without ETL (3.4%).

**Figure 19:**
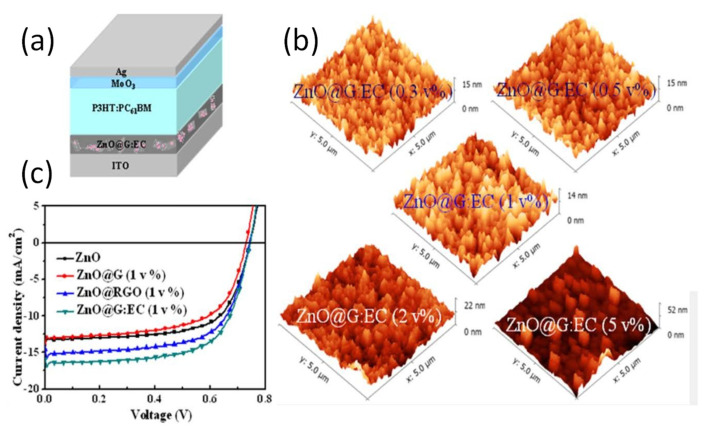
(**a**) Depiction of ITO/ZnO@G:EC/P3HT:PC_61_BM/MoO_3_/Ag cell; (**b**) atomic force microscope (AFM) images of ZnO@G:EC nanocomposites with several graphene concentrations; (**c**) *J–V* plots of OSCs with the nanocomposites. Reproduced from ref. [100], with permission from the American Chemical Society.

Another strategy is the doping of graphene with zinc nanorods (ZnR [104]), which are good electron transport layers for PTB7:PCBM-based OSCs. The cell, with structure (ITO)/ZnR-doped G/PTB7:PCBM/C60-bis/Ag, exhibited a PCE of 7.86%, along with a Jsc of 16.20 mA cm^−2^, one of the highest for OSCs based on GRMs. A novel ETL based on ZnO nanostructures embedded in a graphene matrix with ethyl cellulose (EC) as the stabilizing agent has also been developed [100]. The ZnO@G:EC nanocomposites with different graphene concentrations showed an even morphology, as revealed by AFM images (Figure 19), and preserved the initial structure of graphene as well as its outstanding conductivity. The cell comprising P3HT:PC_61_BM as the active layer, with an architecture of ITO/ZnO@G:EC/P3HT:PC_61_BM/MoO_3_/Ag, exhibited a PCE of 3.9%, around 18 and 27% higher than that with rGO or pristine graphene alone, respectively. By substituting the active layer by PTB7:PC_71_BM, the efficiency increased up to 8.4%, the highest reported for GRMs as the ETL. This easy and versatile strategy can result in highly conductive electron-transport layers for OSCs.

## 4. Stability of GRMs-Based OSCs

For commercial applications, the long-term stability of OSCs against air, humidity, temperature, and light illumination has a huge implication because these cells are damaged more rapidly under atmospheric conditions than the corresponding inorganic counterparts [105]. The durability can be assessed from the standpoint of chemical stability based on materials in the air, degradation caused by reactions with water and oxygen molecules, and the likely degradation of electrode materials. Previous works [106] reviewed the stability of OSCs and concluded that degradation comes from the separation of donor–acceptor phases, as well as the photo-oxidation of active layers and donor materials due to the diffusion of water and oxygen molecules through the interlayer/electrode. Besides, the interface between the HTL and the ITO anode can result in failure. The incorporation of carbon nanomaterials can overcome these problems due to their barrier properties since they can prevent diffusion of atmospheric components into the device layers [107]. Recent studies have reported the long-term stability of GRM-based OSCs in terms of PCE changes as a function of time, and the most relevant results are summarized in Table 5. 

**Figure 20:**
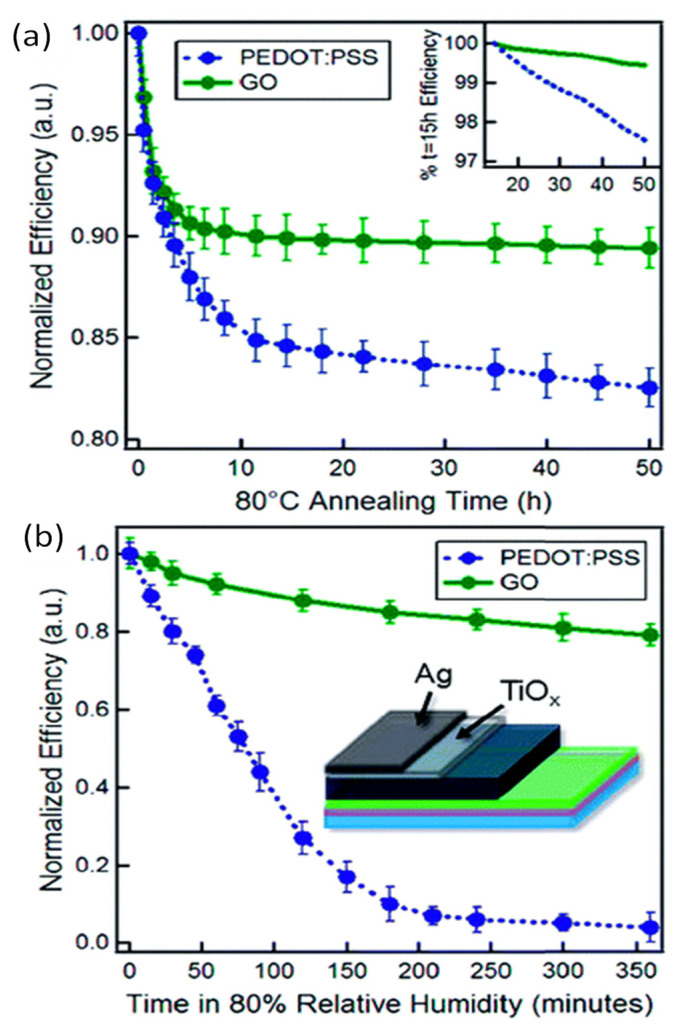
Long-term stability of PTB7:PC_71_BM-based OSCs with conventional PEDOT:PSS and GO interfacial layers. (**a**) Variation of normalized PCE displaying thermal degradation of encapsulated cells at 80 °C under a nitrogen atmosphere. (**b**) Variation of normalized PCE showing environmental degradation of unencapsulated cells under 80% RH. Reprinted from ref. [76], with permission from the American Chemical Society.

PEDOT:PSS has been widely used as interfacial material in OSCs. However, its acidity, morphological phase segregation, and hygroscopic character provoke low stability or even full cell failure. Thus, reduction efficiencies ranging from about 60% [103] to almost 100% [105] have been reported. The total or partial replacement of the PEDOT:PSS layer by GO or its derivatives significantly improves the durability of OSCs, leading to smaller reductions in efficiency (i.e., in the range of 16–29%, Table 5). Figure 20 compares the stability of PTB7:PC_71_BM OSCs with conventional PEDOT:PSS and GO interfacial layers in terms of changes of normalized PCE due to thermal (a) and environmental (b) degradation, with and without encapsulation [76]. Clearly, PEDOT:PSS-based cells degrade much faster, resulting in an almost complete loss of efficiency, while GO-based ones display considerably slower degradation, with an almost 5-fold improvement in PCE retention. A lifetime of 1428 h for PEDOT:PSS and 7156 h for GO-based devices was predicted at 80 °C. At 80% RH, GO-based cells also showed longer retention of PCE: a durability of approximately 6 and 122 h for PEDOT:PSS and GO-based cells, respectively, was predicted. Since PEDOT:PSS is hygroscopic, it attracts water molecules that cause instability and lead to degradation under high humidity. The GO layer can act as a barrier, which prevents the diffusion of oxygen and water molecules from the ITO electrode to the active layers. Overall, the use of a thermally stable and mechanically strong GO layer instead of PEDOT:PSS significantly improves the stability of organic solar cells. 

Other authors [90] used cesium carbonate (Cs_2_CO_3_)-functionalized GQDs as the ETL and investigated their durability. The *V*_oc_ of the GQDs–Cs_2_CO_3_ device was 99.4% of its initial value after 1200 h under atmospheric conditions, whereas the *V*_oc_ of cells comprising only Cs_2_CO_3_ dropped quickly with time. Both types of devices showed a similar degradation trend of *J*_sc_. The GQDs–Cs_2_CO_3_-based solar cells showed a 56% higher PCE and 200% improvement in stability compared to those comprising only Cs_2_CO_3_. The PCE of GQDs–Cs_2_CO_3_ cells was 70% of its initial value upon 450 h under air at RT and 60% RH; the stability was improved 2-fold compared to Cs_2_CO_3_-based devices, since the GQDs provide hole-blocking ability and electron transfer capability, hence preventing the diffusion of the Cs^+^ ion into the active layer/polymer interface. The stability of GONR-based OSCs compared to a reference PEDOT:PSS device after storage under nitrogen atmosphere was also compared [91]. The PCE of the ITO/PEDOT:PSS-based device decreased by 75% after 90 days, whereas the GONR-based counterpart retained about 86% of its initial value, indicating that GONR as an HTL improves environmental stability.

## 5. Conclusions

In this review, the most representative advances using GRMs as electrodes, active layers, and transport layers in OSCs have been summarized. Graphene and its derivatives, GO, rGO, GNRs, GQDs, etc., have arisen as ideal nanomaterials for OSCs due to their nanoscale dimensions, large specific surface area, and superior stiffness combined with flexibility, exceptional electrical conductivity, and high transparency. Despite that the PCEs obtained in GRM-based devices have not gone beyond cells using conventional materials, researchers in the field have already recognized the key features to increase their performance. Lowering GRM sheet resistance, for instance, by increasing the film thickness, would increase the overall PCE. Thickness adjustment also has an important effect on the film transparency. Improved transparency would result in enhanced light transmission and better efficiency. Thus, an optimal equilibrium between transparency and conductivity has to be attained. Thermal treatments (i.e., annealing) are also crucial for the final efficiency; through this easy means, noteworthy PCE increases have been attained. Numerous routes to synthesize GRMs have been proposed (i.e., adhesive tape, epitaxial growth, CVD, liquid-phase exfoliation, etc.). In particular, devices with CVD graphene show improved properties ascribed to its homogeneity and high electronic quality, while graphene produced by exfoliation with solvents has poorer properties. Nevertheless, most of these approaches are not appropriate for cost effective industrial applications. The cell manufacture is another issue. Thus, the development of uniform films as well as the complete control over the interfacial properties between the materials applied in the cell is crucial. In this regard, solution-processable GRMs are preferred due to their solubility in common solvents. In particular, functionalized graphene is the most used GRM material in OSC devices due to its improved processability in solution. A better solubility would guarantee a good interfacial contact that would promote electronic interactions, fostering charge generation. Besides, the graphene concentration is a crucial parameter to take into account, which has to be sufficiently high to develop an interpenetrating donor/acceptor interface and an effective carriage path for the active layer. However, high graphene concentrations typically lead to agglomerates that result in detrimental performance. 

GO-based materials are amongst the best alternatives for PEDOT: PSS, the most common HTL material in spite of its acidic and hygroscopic characteristics that strongly limit the cell durability. A foremost benefit of GO-based materials is that they can be directly assembled from aqueous dispersions and lead to PCE data equivalent to those of cells manufactured with PEDOT: PSS; besides, they show significantly improved long term stability. Thus, the GO layer can act as a barrier, preventing the diffusion of oxygen and water molecules from the ITO electrode to the active layers. In addition, the release of oxygen from the GO sheet promotes the conversion from insulator to semiconducting behavior. Therefore, approaches to obtain functionalized GO using non-toxic chemicals is one of the chief tendencies in this research arena. Regarding ETL materials, one of the key aims is to attain highly conductive materials with a low work function to enhance electron mobility, which can be achieved by doping graphene with different atoms. The highest PCE (about 15%) has been recently attained via a combination of a polymer with CVD-graphene as the transparent electrode. Novel functionalized GRMs should be developed to match the energy levels of traditional donor and acceptor materials in the active layer and the transparent electrodes. It is expected that constant investigation struggles in this emergent area would result in a flourishing field of photovoltaic technologies.

## Figures and Tables

**Table 1:** PV parameters for OSCs incorporating GRMs electrodes.

Cell Configuration	*J_sc_* (mA·cm^−2^)	*V_oc_* (V)	FF (%)	PCE (%)	Ref.
ITO/ZnO/P3HT:PC_61_BM/PEDOT: PSS + Au/CVD-G (1L)	10.58	0.59	43.0	2.70	[39]
PMMA-GF/ PEDOT:PSS /n-Si/LiF/Al	25.08	0.55	39.0	5.48	[40]
GF/PEDOT:PSS/P3HT:PCBM/Al	3.44	0.55	29.0	0.68	[41]
Ag-G/)/PEDOT:PSS/P3HT:PCBM/Al	9.20	0.58	38.00	2.00	[42]
CVD-G (1L)/PEDOT:PSS + Au/P3HT:PCBM/Ag	10.78	0.60	45.7	2.91	[43]
CVD-G (2L)/PEDOT:PSS + Au/P3HT:PCBM/Ag	10.61	0.60	50.0	3.17	[43]
CVD-G (4L)/PEDOT:PSS + Au/P3HT:PCBM/Ag	9.88	0.57	49.7	2.80	[43]
G/PEDOT:PSS/PTB7-F40: PC_71_BM/TiOx/Al	12.9	0.71	65.0	6.00	[44]
CVD-G/ZnO/PTB7:PC_71_BM/MoO_3_/Ag	14.1	0.72	69.5	7.10	[45]
CVD-G/PEDOT:PSS/MoO_3_/PTB7:PC_71_BM/Ca/Al	14.4	0.72	58.7	6.10	[45]
G/PEDOT:PSS/P3HT:PC_61_BM/LiF/Al	9.20	0.58	57.0	3.00	[46]
G-TFSA/MoS_2_/ P3HT:PCBM/Al	10.01	0.58	60.94	3.19	[47]
Au-GNRs/PEDOT:PSS-WO_3_/ PTTBDT-FTT:PC_71_BM/Ca/Al	8.45	1.56	64.3	8.48	[48]
ITO/ PEDOT:PSS/ PV-D:PCBM/GO-Ag	7.20	0.78	58.0	2.60	[49]
G/PTB7:PC_71_BM/MoO_3_/Ag	10.11	0.25	27.6	0.58	[50]
G-Cu/PTB7:PC_71_BM/MoO_3_/Ag	12.99	0.58	42.0	2.96	[50]
G/PEDOT:PSS/PTB7:PC_71_BM/Al	14.40	0.53	41.8	3.19	[50]
G-Cu/PEDOT:PSS/PTB7:PC_71_BM/Al	13.01	0.73	45.9	4.12	[50]
EG/PEDOT:PSS/PTB7:PC_71_BM/Ba /Al	6.52	0.71	0.56	4.23	[51]
PMMA-G/MoO_3_/PEDOT:PSS /PCDTBT:PC_70_BM/Ca/Al	8.88	0.83	0.45	3.30	[52]
CVD-BrG/PEDOT:PSS/P3HT:ICBA/TiOx /Al	6.90	0.76	34.0	2.10	[53]
Au-G/Al-TiO_2_/P3HT:PCBM/MoO_3_/Ag	8.55	0.60	50.1	2.58	[54]
G/PEDOT:PSS/P3HT:PCBM/TiOx/Al	9.03	0.60	0.48	2.58	[55]
G/PEDOT:PSS/P3HT:PCBM/Al	10.2	0.62	25.2	3.98	[56]
GQDs-doped G-Ag NWs/PEDOT:PSS/P3HT:PCBM/Al	10.43	0.59	59.1	3.66	[57]
AgNW-G/ZnO/PTB7:PC_71_BM/MoO_3_/Ag	15.83	0.69	60.0	6.47	[58]
G-AgNWs/PH1000/PEDOT:PSS/ PM6:Y6/PDINO/Al	23.20	0.83	69.8	13.44	[59]
AZO-G-Cu-AZO/PEDOT:PSS /P3HT:PCBM/PCBM/Ca/Al	10.87	0.63	67.0	4.63	[60]
PI-GF/PEDOT: PSS/PM6:Y6/PDINO/Al	25.8	0.84	70.0	15.2	[61]

GF: graphene film; EG: electrochemically exfoliated graphene; BrG: brominated graphene; 1L: monolayer graphene; 2L: double-layer graphene; 4L: few layer graphene: Ag NWs: silver nanowires; PI: polyimide; Al-doped ZnO (AZO); TFSA: bis(trifluoromethanesulfonyl)-amide.

**Table 2:** PV parameters for OSCs incorporating GRMs in the active layer.

Cell Configuration	*J_sc_* (mA·cm^−2^)	*V_oc_* (V)	FF (%)	PCE (%)	Ref.
G-TFSA/MoS_2_/P3HT:PCBM-GQDs/Al	10.88	0.58	66.1	4.23	[47]
ITO/PEDOT:PSS/P3OT:G/LiF/Al	4.20	0.92	37.0	1.40	[62]
ITO/PEDOT:PSS/P3HT:G/LiF/Al	4.00	0.72	38.0	1.10	[63]
ITO/ZnO/P3HT/PEDOT:PSS/Ag	0.37	0.02	21.0	0.18	[64]
ITO/ZnO/P3HT:rGO/PEDOT:PSS/Ag	0.42	6.18	37.0	0.70	[64]
ITO/ZnO/P3HT:PCA-g-rGO/PEDOT:PSS/Ag	0.48	6.81	33.0	1.17	[64]
ITO/ZnO/P3HT:PCA-rGO/GO/Ag	0.49	8.25	31.0	1.35	[64]
ITO/ZnO/PCA-g-rGO/P3HT:PCA-g-rGO/GO/Ag	0.56	12.09	41.0	2.85	[64]
ITO/ZnO/PTB7-Th-GNRs/MO_3_/Al	15.1	0.80	62.9	7.60	[65]
ITO/PEDOT:PSS/P3HT:EG/Al	10.7	0.65	52.7	3.75	[66]
ITO/PEDOT:PSS/P3OT:PCBM–G/LiF/Al	4.60	0.67	37.1	1.14	[67]
ITO/PEDOT:PSS/P3HT-f-MWCNTs-SPFG/LiF/Al	4.70	0.67	32.0	1.05	[68]
ITO/PEDOT:PSS/G-g-P3HT:C_60_/Al	3.50	0.43	41.0	0.61	[69]
ITO/PEDOT:PSS/C_60_-g-rGO:P3HT/Al	4.45	0.56	49.0	1.22	[70]
ITO/PEDOT:PSS/P3HT:GQDs/Al	6.33	0.67	30.2	1.28	[71]
ITO/PEDOT:PSS/P3HT/ANI-GQDs/Al	3.51	0.61	53.0	1.14	[72]
ITO/PEDOT:PSS/P3HT:PCBM:GQDs/LiF/Al	26.46	0.60	33.0	5.24	[73]
ITO/PEDOT:PSS/rGOQDs:PTB7: PC71BM/Al	15.20	0.74	67.6	7.60	[74]
ITO/PEDOT:PSS/GOQDs:PTB7: PC71BM/Al	16.10	0.73	60.4	7.11	[74]
ITO/PEDOT:PSS/N-doped G:P3HT:PCBM/Al	14.95	0.59	51.0	4.48	[75]
ITO/PEDOT:PSS/GO:PTB7: PC_71_BM/LiF/Al	15.21	0.71	67.7	7.39	[76]
ITO/PEDOT:PSS/P3HT:PCBMrGO-GeO_2_ /LiF/Al	17.10	0.44	31.0	2.30	[77]
ITO/PEDOT:PSS/PTB7:PCBM:SPFG/PFN/FM	17.27	0.77	50.58	6.45	[78]
ITO/PEDOT: PSS/P3HT:PC_60_BM:GO/LiF/Al	18.20	0.57	43.0	4.40	[79]
ITO/ZnO/PTB7:PC_71_BM:P-GO/MoO_3_/Ag	14.69	0.74	67.3	7.52	[80]
ITO/PEDOT:PSS/PTB7-th:PCBM:F-rGO/LiF/Al	12.10	0.77	79.0	7.36	[81]

ANI-GQDs: Aniline-functionalized graphene quantum dots functionalized multiwalled carbon nanotubes: f-MWCNTs. PCA-g-rGO: 1-pyrenecarboxylic acid-grafted-GO; SPFG: solution processable functionalized graphene; FM: Bi/In/Sn; P-GO: graphene oxide functionalized with conjugated polymer.

**Table 3:** PV parameters for OSCs incorporating GRMs as the HTL.

Cell Configuration	*J_sc_* (mA·cm^−2^)	*V_oc_* (V)	FF (%)	PCE (%)	Ref.
ITO/rGO/PTB7:[70]PCBM/PFN/FM	13.43	0.70	58.0	5.50	[78]
ITO/GO/PCDTBT:PC70BM)/LiF/Al	8.97	0.83	54.0	4.04	[79]
ITO/GO/AuNPs/P3HT:PCBM/Al	10.05	0.82	58.3	3.37	[82]
ITO/GO/PEDOT:PSS/PCDTBT: PC71BM/Al	10.44	0.82	50.0	4.28	[83]
ITO/PEDOT:PSS(Au@PEG–GO) /PBDTTT-CT:PC_71_BM/Ca/Al	16.1	0.75	60.0	7.26	[84]
ITO/PRGO/PEDOT:PSS/PTB7-th:PBCM/Al	14.78	0.76	64.5	7.24	[85]
ITO/ZnO/C60-SAM/P3HT:PCBM/GO/Al	8.78	0.64	64.0	3.60	[86]
ITO/GO/P3HT:PCBM/Al	11.4	0.57	54.0	3.5	[87]
ITO/FLGs-PEDOT:PSS/PCBM/P3HT/Ca/Al	9.44	0.58	55.0	3.7	[88]
ITO/GO/PEDOT: PSS/P3HT: PC60BM/ZnO/Au	15.42	0.49	64.2	4.82	[89]
ITO/GQDs–Cs_2_CO_3_/P3HT:PCBM/V_2_O_5_/Au	8.95	0.58	58.5	3.05	[90]
ITO/GONRs/P3HT:PCBM/Al	9.96	0.62	67.0	4.14	[91]
ITO/GO/P3HT:PCBM/Al	8.42	0.62	60.0	3.28	[91]
ITO/spr-GO/P3HT:PCBM/Al	9.33	0.59	66.7	3.63	[92]
ITO/GO-OSO3H/P3HT:PCBM/Al	10.15	0.61	71.0	4.37	[93]
ITO/GO*/P3HT:PCBM/Al	10.38	0.57	62.6	3.98	[94]
ITO/rGO/PEDOT: PSS/P3HT: PCBM/Al	10.04	0.59	65.9	3.94	[94]
ITO/GO-SWCNT/P3HT:PCBM/Ca/Al	10.82	0.60	62.8	4.10	[95]
ITO/F-rGO/PTB7:PC71BM/PFN/FM	13.55	0.68	57.3	5.73	[96]
ITO/F-rGO/PTB7:PC71BM/PEN/Al	16.89	0.79	65.0	8.60	[97]
ITO/F-rGO/PBDB-T:ITIC/PFN/FM	13.40	0.67	58.0	5.30	[98]
ITO/GO/P3HT:PCBM/Ca/Al.	9.65	0.60	58.03	3.36	[99]
ITOr/GO-GO/P3HT:PCBM/Ca/Al.	10.37	0.60	67.66	4.21	[99]

C60-SAM: fullerene derivative with pyrrole and benzoic acid; spr-GO: solution-processable reduced graphene oxide; GO*: thermally treated GO; GONR: graphene oxide nanoribbon; F-rGO: fluorinated reduced graphene oxide; PRGO: polyacrylonitrile-grafted rGO; SCWNT: single-walled carbon nanotubes.

**Table 4:** PV parameters for OSCs incorporating GRMs as the ETL.

Cell Configuration	*J_sc_* (mA·cm^−2^)	*V_oc_* (V)	FF (%)	PCE (%)	Ref.
ITO/ZnO@G/P3HT:PC_61_BM/MoO_3_/Ag	6.57	0.59	63.0	2.40	[100]
ITO/ZnO@rGO/P3HT:PC_61_BM/MoO_3_/Ag	7.91	0.63	67.0	3.30	[100]
ITO/ZnO@G:EC/P3HT:PC_61_BM/MoO_3_/Ag	7.73	0.63	73.0	3.90	[100]
ITO/ZnO@G/PTB7:PC_71_BM/MoO_3_/Ag	13.76	0.73	63.0	6.20	[100]
ITO/ZnO@rGO/PTB7:PC_71_BM/MoO_3_/Ag	14.49	0.74	59.0	7.60	[100]
ITO/ZnO@G:EC/PTB7:PC_71_BM/MoO_3_/Ag	15.88	0.74	68.0	8.40	[100]
ITO/GO/P3HT:PCBM/GO-Cs/Al	10.30	0.61	69.0	3.67	[101]
ITO/GO-Cs/P3HT:PCBM/GO/Al	10.69	0.51	54.0	2.97	[101]
ITO/PEDOT:PSS/P3HT:PCBM/ rGO-pyrene-PCBM/Al	9.07	0.64	62.1	3.39	[102]
ITO/PEDOT:PSS/PCDTBT:PCBM/GO-TiOx/Al	12.40	0.88	68.0	7.50	[103]
ITO/PEDOT:PSS/PCDTBT:PCBM/GO/Al	12.36	0.86	63.0	6.72	[103]
(ITO)/ZnR-doped G/PTB7:PCBM/C60-bis/Ag	16.20	0.75	64.69	7.86	[104]

**Table 5:** Comparison of the initial PCE and PCE after degradation for GRM-based OSCs.

Cell Configuration	Initial PCE (%)	PCE after Degradation (%)	Degradation Conditions	Ref.
G-TFSA/MoS_2_/P3HT:PCBM/Al	3.19	2.23	500 h, air	[47]
G-TFSA/MoS_2_/P3HT:PCBM-GQDs/Al	4.23	3.38	500 h, air	[47]
ITO/PEDOT:PSS/P3HT:EG/Al	3.75	3.02	42 h, air	[66]
ITO/PEDOT:PSS/GO:PTB7: PC_71_BM/LiF/Al	7.39	5.92	6h, 80% RH	[76]
ITO/PEDOT:PSS/PTB7: PC_71_BM/LiF/Al *	7.46	0.38	6h, 80% RH	[76]
ITO/GQDs–Cs_2_CO_3_/P3HT:PCBM/V_2_O_5_/Au	3.05	2.13	450 h RT, 65% RH	[90]
ITO/GONRs/P3HT:PCBM/Al	4.14	3.56	2160 h, N_2_	[91]
ITO/PEDOT:PSS/P3HT:PCBM/Al *	3.08	2.31	2160 h, N_2_	[91]
ITO/rGO/PEDOT: PSS/P3HT: PCBM/Al	3.94	2.76	48 h, 350 °C	[94]
ITO/GO/P3HT:PCBM/Ca/Al.	3.36	2.69	8h, RT, 80% RH	[99]
ITOr/GO-GO/P3HT:PCBM/Ca/Al.	4.21	3.07	8h, RT, 80% RH	[99]
ITO/PEDOT:PSS/PCDTBT:PCBM/GO-TiOx/Al	7.50	7.24	720 h, air	[103]
ITO/PEDOT:PSS/PCDTBT:PCBM/GO/Al	6.72	6.52	720 h, air	[103]
ITO/PEDOT:PSS/ PCDTBT:PCBM/Al *	5.23	2.32	720 h, air	[103]

* Reference cell.

## Data Availability

Data sharing is not applicable to this article as no new data were created or analyzed in this study.
